# Comparative evaluation of enamel microhardness after using two different remineralizing agents on artificially demineralized human enamel: An *in vitro* study

**Published:** 2020-09-05

**Authors:** J. Sai Sahiti, N. Vamsee Krishna, S. Datta Prasad, C. Sunil Kumar, S. Sunil Kumar, K. S. Chandra Babu

**Affiliations:** Department of Conservative Dentistry and Endodontics, CKS Theja Institute of Dental Sciences and Research, Tirupathi, Andhra Pradesh, India

**Keywords:** Tricalcium phosphate, hydroxyapatite, fluoride, remineralization, microhardness

## Abstract

**Aim:**

The purpose of this study was to compare the remineralization outcomes of two agents using the Vickers microhardness test (VMHT) on artificially induced enamel subsurface lesions.

**Materials and Methods:**

Forty sound extracted premolars were selected as samples for the current study and divided into four groups of 10 teeth each: Clinpro (group 1), Remin Pro (group 2), untreated positive control (group 3), and a demineralized negative control (group 4). All the sample groups were assessed first at baseline then after demineralization and remineralization using DIAGNOdent. After the remineralization process, VMHT was performed on all sample groups to assess surface microhardness (SMH). The results obtained were then compared using a one-way analysis of variance (ANOVA) for the difference in SMH.

**Results:**

Vickers microhardness number values were analyzed using one-way ANOVA and samples in the experimental groups 1 and 2 showed a statistically significant difference compared to the control groups (*P* < 0.05). Remineralization was higher in the Remin Pro group, followed by Clinpro group.

**Conclusions:**

The results of this *in vitro* study show that both Remin Pro and Clinpro are equally effective as remineralizing agents. Although Remin Pro tended to yield a higher microhardness, no significant differences were observed between the two agents.

**Relevance for Patients:**

Enamel mineral loss leads to the degradation of the surface and subsurface structures of teeth. Natural remineralization that occurs physiologically is not sufficient to overcome the hurdles of mineral loss a tooth undergoes due to changes in food habits and lifestyle. A thought on remineralization and management such as prescribing remineralizing agents and regular professional topical fluoride applications would definitely render satisfactory results by a strong healthy enamel.

## 1. Introduction

Remineralization of dental enamel is the reconstitution of the lost mineral content in teeth. Prevention strategies maximize the conservation of dental tissue by promoting mineralization and resistance to conditions conducive to demineralization and dental cavities. Clinical tooth demineralization management should concentrate on early detection and prevention strategies, that is, remineralization of the tooth before dental tissue degradation becomes irreversible and results in permanent loss of dental tissue and formation of a cavity. Demineralization is a process caused by dental plaque acids, which dissolve the organic and inorganic content of tooth minerals that make up the basic calcium, phosphate, and hydroxyl crystals found in enamel, dentin, and cementum. Demineralization refers to decreased mineral content that in turn, decreases the hardness of the tooth and marks the necessity of remineralization. Remineralization requires the availability of the same ions to rebuild the missing or damaged rods ideally with fluoride as a catalyst.

Demineralization is damage to tooth enamel integrity that can be stopped and/or reversed. Evaluating the presence of demineralization can be achieved using a DIAGNOdent device, an optical laser-based fluorescence instrument that detects demineralized carious lesions by analyzing the intensity of reflected fluorescence from tooth structure [1]. Sound tooth structure reflects a lower fluorescence intensity signal compared to demineralized or carious lesions [2,3]. Aggravation or reversal of lesions depends on the balance between demineralization – supporting pathological factors (cariogenic bacteria, dysfunction of saliva, and fermentable carbohydrates) and protective factors (antibacterial agents, remineralizing ions, and sufficient saliva) that tip the balance towards remineralization [4]. Dental remineralization technology has advanced with combinations such as fluoride, hydroxyapatite (HA), and xylitol, ingredients used in Remin Pro (VOCO GmbH, Cuxhaven, Germany), which is believed to be associated with calcium in aqueous solution to inhibit the dissolution of calcium and/or phosphate ions from enamel and to act as a carrier of calcium required for enamel remineralization [5]. HA is a major mineral component found in the dental matrix and is what gives teeth its rigidity. Clinpro (3M ESPE, Pymble, New South Wales, Australia) is an anticaries tricalcium phosphate (beta-TCP) and sodium fluoride (NaF) dentifrice. The combination of fluoride with beta-TCP provides greater remineralization in terms of fluoride absorption and microhardness [6]. Tooth structure microhardness can be measured a technique known as the Vickers microhardness test (VMHT) [7]. VMHT uses a diamond indent area to impress a small area on the tooth surface with a predefined set load for a specified amount of time. The microhardness number is later computed after microscopic examination of the indentation in relation to the used indentation load and the area of the remaining impression.

The aim of this study was to evaluate and compare enamel microhardness after using two different remineralizing agents in an *in vitro* demineralization model on human tooth enamel.

## 2. Materials and Methods

The research protocol and study guidelines were reviewed and approved by the Scientific Review Committee of CKS Teja Institute of Dental Sciences and Research (Ref. No. CKS/ENDO/18-19/005), Tirupathi, Andhra Pradesh, India.

### 2.1. Study specimens

A total of 60 recently extracted sound premolar teeth were collected from the Department of Oral and Maxillofacial Surgery. Forty teeth were selected based on the following criteria for inclusion: Sound tooth structure free from cracks, restorations, stains, white spot lesions, or caries evident by naked eye and teeth extracted for orthodontic purposes. Exclusion criteria included teeth with enamel hypoplasia or any other developmental anomalies, teeth with any visible or detectable caries, and teeth showing a DIAGNOdent score of more than 7 were excluded from the study.

### 2.2. Preparation of the demineralizing solution

A demineralizing solution was prepared in the Department of Biochemistry of the Krishna Teja Pharmacy College in Tirupathi. A digital pH meter (Equinox microprocessor-based benchtop pH meter, model no: 86501, Valli Aqua and Process Instruments, Tamil Nadu, India) was used to check pH during and after preparation of the demineralizing solution. Each time before checking pH, the instrument was calibrated using a phosphate buffer solution of pH = 7.0. The composition of demineralizing solution used was 2.2 mM calcium chloride (CaCl_2_.2H_2_O), 2.2 mM monosodium phosphate (NaH_2_PO_4_.7H_2_O), and 0.05 M lactic acid [8]. The pH was adjusted to 4.5 with 50% sodium hydroxide (NaOH).

### 2.3. Procedure of demineralization and study groups

Each tooth was coated with nail varnish, leaving an enamel window of 3 × 3 mm on the buccal surface in the middle one-third of the crown. From 40 premolar specimens, 30 were randomly selected and immersed into a glass container containing 50 mL of demineralizing solution at room temperature for a period of 72 h. The demineralizing procedure was intended to produce a consistent subsurface lesion. After 72 h in the demineralizing solution, the teeth were washed with deionized water and dried with the help of an air syringe.

The demineralized samples were evaluated with a DIAGNOdent instrument (KaVo, Biberach, Germany) to determine the presence of a subsurface lesion on the tooth surface. A measurement registering a value of 9 and above confirmed the presence of a subsurface lesion. The DIAGNOdent instrument was calibrated against its own ceramic standards according to manufacturer recommendations before every measurement session; a type B probe [8] was used in the study. After confirming demineralization with the DIAGNOdent, the 30 demineralized samples were randomly divided into three equal groups of 10 each: TCP (Clinpro Tooth Crème, 3M ESPE, Pymble, New South Wales, Australia) (group 1), fluoride, HA, xylitol (Remin Pro, VOCO GmbH, Cuxhaven, Germany) (group 2), and demineralized negative control (group 4). The remaining 10 untreated premolar specimens were used as positive controls (group 3).

### 2.4. Procedure of remineralization

The samples in groups 1 and 2 were treated with respective remineralizing agents at every 24 h for 7 days with the help of a cotton applicator tip for 4 min, washed with deionized water, and placed in artificial saliva. A commercially available artificial saliva solution (wet mouth saliva substitute, Apexion Dental Products & Services, Kerala, India) was used and consisted of a mixture of 0.4 g of sodium chloride (NaCl), 0.4 g of potassium chloride (KCl), 0.795 g of calcium chloride (CaCl_2_.H_2_O), 0.69 g of sodium dihydrogen phosphate (NaH_2_PO_4_.H_2_O), 0.005 g of sodium sulfide (Na_2_S.9H_2_O), and 1000 mL of distilled water [9]. Artificial saliva was changed every 24 h just before immersion of freshly treated samples. After 7 cycles of remineralization, the surface was assessed for remineralization changes using DIAGNOdent; the samples from experimental groups showed a moment value between 3 and 5. All 40 samples were mounted in acrylic blocks and then subjected to a standardized VMHT (Highwood DMH 7, Model HWMMT-X7, TTS Unlimited, Osaka, Japan) under 200 g load for 15 s [7,10].

### 2.5. Statistical analysis

Using one-way analysis of variance (ANOVA) for comparing mean surface microhardness (SMH) between 4 different groups, a sample size calculation with an estimated effect size of 0.60 with an alpha of 5% and a power of 85% results in a total sample size of 40 (n = 10 per group). SPSS program (IBM SPSS Statistics, IBM, Armonk, NY, USA) was used to calculate descriptive statistics. Data were analyzed for intergroup comparison using one-way ANOVA. Individual pairwise comparison was performed using a post hoc least significant difference (LSD) test. Statistical significance was considered when *P* < 0.05.

## 3. Results

There was a statistically significant overall group interaction in mean microhardness between the four groups (*P* = 0.030) (Table 1). There was no statistically significant difference between groups 1 and 2, 1 and 3, and 2 and 3 in an individual pairwise comparison. Post-hoc LSD tests revealed significant differences between all the study groups (1, 2, and 3) when compared with group 4 (demineralized negative control) (Table 2) (*P* < 0.001).

**Table 1:** Overall intergroup comparison of microhardness (one-way ANOVA)

Group	N	Min.	Max.	Mean	SD	F-value	*P*-value
Group 1	10	238.5	330.3	281.63	27.23	3.3	0.030*
Group 2	10	228.4	344.7	290.01	37.27	
Group 3	10	263.2	311.4	290.72	16.36	
Group 4	10	233.8	276.9	257.77	13.61	

* Significant (*P*<0.05); group 1: Clinpro, group 2: Remin Pro, group 3: Untreated positive control, group 4: Demineralized negative control

**Table 2:** Individual pairwise comparison of microhardness of all the groups

Intergroup comparison		Mean difference	Std. deviation	Std. error	Sig.
Group 1	Group 2	−8.38	58.314	1.82067	0.326
	Group 3	−9.09	32.330	1.82067	0.411
	Group 4	23.86	23.454	1.82067	0.016*
Group 2	Group 3	−0.71	42.046	1.15149	0.852
	Group 4	32.24	40.911	1.15149	<0.001**
Group 3	Group 4	32.95	21.573	1.15149	<0.001**
		**The mean difference is significant at the 0.001 level.				
		*The mean difference is significant at the 0.05 level.				

Group 1: Clinpro, Group 2: Remin Pro, Group 3: Untreated positive control, Group 4: demineralized negative control

## 4. Discussion

The present study was undertaken to evaluate the remineralization outcome associated with two remineralizing agents in an early enamel demineralization model using a DIAGNOdent and VMHT. The *in vitro* demineralization model successfully achieved demineralization in the isolated specimens. The results showed that both remineralizing agents were able to yield similar levels of microhardness following the remineralizing procedures. Remin Pro showed a slightly higher level in microhardness in comparison Clinpro although this was not statistically significant.

The balance between demineralization and remineralization is largely mediated by the acidic microenvironment intraorally, produced by bacteria, and the buffering capacity of saliva in neutralizing acidic pH levels. A drop in pH of the oral cavity results in tooth demineralization. Conversely, a rise in pH results in the deposition of calcium, phosphate, and fluoride, hence a reversal of the demineralization process. To prevent demineralization effectively, early intervention is essential and possible through the application of commercially available preparations consisting of, for example, fluoride, calcium, phosphate-based systems, and calcium sodium phosphosilicate. Considering the significance of the enamel surface layer in caries progression, the assessment of changes in this region is relevant. To this end, SMH measurement is a useful technique that can be used to determine enamel surface hardness. SMH indentation provides a proportionately simple and abrupt method in demineralization and remineralization studies in materials of fine microstructure, non-homogenous, or prone to cracking such as dental enamel [11]. DIAGNOdent was used in this investigation to assess demineralization and remineralization and VMHT was used to measure tooth surface hardness.

DIAGNOdent laser fluorescence is a non-invasive method used to measure early demineralization in teeth [8,12]. The surface of the tooth absorbs the laser light and emits fluorescence in the infrared spectrum field. As the laser light enters and penetrates into the site of interest, two-way handpiece optics allow the device to simultaneously quantify the reflected laser light energy. Using a 655-nm wavelength, intact healthy tooth structure exhibits little or no fluorescence resulting in very low-scale display readings. Demineralized areas will, however, exhibit fluorescence proportionate to the degree of demineralization resulting in elevated scale readings. Since it is considered a safe and non-invasive technique, values can be obtained repeatedly and compared, rendering the DIAGNOdent an effective real-time chairside instrument to appraise subsurface lesions associated with demineralized enamel [13]. Enamel may appear to look healthy and caries free, but caries lesions are not the only reasons for damaged or compromised enamel surfaces. White spot lesions, initial lesions and cracks can be detected with DIAGNOdent, showing equally elevated fluorescence levels that can be mistaken for demineralization result in false positives [2].

Microhardness measurements can be evaluated by two different parameters, Knoop hardness number, and Vickers hardness number. VMHTs are used in most cases to assess the hardness of materials within the range of microhardness test loads (typically 1-1000 g). However, Knoop hardness test is often used when testing the hardness of thin layers such as ceramics or coatings to overcome the problem of cracking in brittle materials. The square shape of the residual indentation obtained by VMHT is easy and accurate to measure under a microscope. Minutest changes in square shape can be easily detected, whereas rhomboid shape indentations with opposing surfaces parallel to each other, obtained with Knoop hardness, make it difficult to detect errors [14]. Values are determined by optically measuring diagonal lengths of the impression left by the indenter. Normally, the Vickers hardness value (HV) is measured by the equation: HV = 1854 . F/d^2^, where the constant value of each equation is determined using the specific geometry of the indenter, “F” is the indentation load (g), and “d” is the diagonal of the indentation (mm) [7].

The remineralizing agent Remin Pro contains fluoride (1450 ppm), HA, and xylitol (antibacterial agent) and has been suggested that it can mitigate dental hypersensitivity, prevent demineralization, and enhance remineralization of subsurface lesions. In the present investigation, VMHT results showed that Remin Pro yielded higher microhardness in comparison to Clinpro. HA provides remineralizing agents with the potential to repair microcracks in enamel, thereby improving the hardness of enamel by HA present in preparations like Remin Pro. According to Miake *et al.*, xylitol can induce remineralization of deeper layers of demineralized enamel by facilitating calcium ion movement and accessibility [5]. Our results are in accordance with Thakur Sandeep *et al*. [14], Kamath *et al*. [15], and Jaya Shankara *et al*. [16]. These studies were in accordance as the studies were similar to the present study on account of remineralization and the remineralizing agent Remin Pro had shown the better remineralization potential compared with other agents used in the studies.

Clinpro is used clinically in a similar way as Remin Pro. Clinpro contains 0.21% NaF (950 ppm fluoride) and facilitates remineralization, prevents demineralization, and caries progression. In this study, Clinpro yielded lower scores under VMHT and our findings are consistent with other studies [17-21]. According to Rao *et al.*, Clinpro crème was noted in a report as a new material obtained using a milling technique incorporating beta TCP and sodium lauryl sulfate or fumaric acid. This idea behind the Clinpro formulation was hypothesized to provide a “functionalized” calcium and a “free” phosphate designed to drive fluoride remineralization effectiveness [6]. BetaTCP mimics apatite structure and possesses unique calcium ions capable of interacting with fluoride and enamel; this hybrid calcium system combines with demineralized enamel to boost benefits associated with fluoride remineralization. When TCP encounters a salivated tooth surface, the protective barrier breaks down, making calcium, phosphate, and fluoride ions available. The fluoride and calcium then react with weakened enamel to initiate and enhance mineral growth in relation to fluoride. This mechanism delivers more calcium and fluoride to the teeth tissue. The purpose of functionalizing beta-TCP is to enable targeted low-dose delivery by creating barriers that prevent premature fluoride-calcium interactions when applied to teeth by means of dentifrices or mouthwashes [1]. However, the process can become “too efficient,” for example, using very high fluoride concentrations or highly-saturated remineralizing solutions; comprehensive remineralization of lesion surfaces can effectively “seal” the subsurface lesion body and further prevent remineralization [22].

In the present study, Remin Pro containing 1450 ppm of fluoride yielded slightly higher remineralization and microhardness in comparison to Clinpro, containing only 950 ppm of fluoride. It is well established that fluoride levels within the 0.01-0.2 ppm range can adequately induce seeded growth of HA. Since the present investigation was conducted in an *in vitro* setting, the oral environment could not be emulated to match exact *in vivo* conditions. However, an important advantage associated with the *in vitro* model presented in this report is the ability to conduct a well-controlled single variable investigation to determine mechanistic components that would otherwise be difficult to scrutinize in a complex *in vivo* setting. More translational research on remineralization of dental tissues is required to identify functional therapeutic targets that would optimize remineralization strategies in preventive dentistry.

## 5. Conclusion

Within the limitations of this study, we may conclude that both Clinpro and Remin Pro may be valuable remineralizing agents that are able to restore dental enamel remineralization and hardness after demineralization. Although Remin Pro showed marginally elevated remineralization (though not significant) in comparison to Clinpro, both agents appear to be equally effective. Future long-term clinical trials should be conducted to determine the superiority of these remineralizing agents in vital teeth.

### Disclosure Statement

The authors declare no potential conflicts of interest with respect to the authorship and/or publication of this article.

### Funding Source

None to declare.
